# Novel markers of human ovarian granulosa cell differentiation toward osteoblast lineage: A microarray approach

**DOI:** 10.3892/mmr.2021.12308

**Published:** 2021-07-21

**Authors:** Maciej Brązert, Wiesława Kranc, Piotr Celichowski, Katarzyna Ożegowska, Joanna Budna-Tukan, Michal Jeseta, Leszek Pawelczyk, Małgorzata Bruska, Maciej Zabel, Michał Nowicki, Bartosz Kempisty

Mol Med Rep 20: 4403-4414, 2019, DOI: 10.3892/mmr.2019.10709

Following the publication of this paper, the authors have requested that, on p. 4412 of the above article in the Funding section of the Declarations, the acknowledgement to one of the funding sources should be removed from the paper; essentially, the reference to grant no. 2018/31/B/NZ5/02475, formulated by the Polish National Science Centre (grant providing institution), should be removed from the paper. Therefore, the revised version of the Funding section paragraph should read as follows:

## Notes

The authors confirm that there are no further errors in the study, and all the authors agree to this correction. The authors are grateful to the Editor of *Molecular Medicine Reports* for granting them this opportunity to publish a Corrigendum, and apologize for any inconvenience caused.

